# Pigmented median raphe cyst of the penis with consideration of the possible mechanism of melanocytic colonization: A case report

**DOI:** 10.3892/ol.2013.1719

**Published:** 2013-11-29

**Authors:** MITSUAKI ISHIDA, MUNEO IWAI, KEIKO YOSHIDA, AKIKO KAGOTANI, HIDETOSHI OKABE

**Affiliations:** Department of Clinical Laboratory Medicine and Division of Diagnostic Pathology, Shiga University of Medical Science, Otsu, Shiga 520-2192, Japan

**Keywords:** median raphe cyst, pigmented, stem cell factor, endothelin-1

## Abstract

Median raphe cyst is a rare lesion located on the median raphe. The cyst wall is lined by cuboidal to columnar cells, transitional (urothelial) cells, stratified squamous cells or a mixture of these. The normal urethral mucosa and the median raphe cyst usually lack melanocytes and/or melanin pigment. However, albeit extremely rare, the presence of melanin pigment and/or melanocytes in median raphe cyst, namely pigmented median raphe cyst, has been previously reported. The current case report presents the sixth case of pigmented median raphe cyst and discusses the possible mechanism of melanocytic colonization in this tumor. A 48-year-old male presented with a nodule on the ventral surface of the penis. Histopathological study revealed that the cyst wall was covered by uniform bland cuboidal to urothelial cells. The peculiar observation was the presence of dendritic melanocytes among the epithelial cells. Therefore, a diagnosis of pigmented median raphe cyst was determined. Immunohistochemically, stem cell factor and endothelin-1 were not expressed in the epithelial cells of the cyst wall. It is well-known that melanocytes are rarely found in various non-melanocytic tumors, a phenomenon termed ‘colonization’. The mechanism by which melanocytes appear in median raphe cyst remains unclear. The present report is the first to demonstrate that melanocytic proliferation and differentiation factors, such as stem cell factor and endothelin-1, are not involved in the pigmentation of median raphe cyst. In addition, aberrant melanocytic migration may contribute to the development of this type of lesion.

## Introduction

Median raphe cyst is a rare benign lesion located in the median raphe and is thought to be derived from the urethral mucosa or urethral gland (Littre’s gland). It appears commonly in children or adolescents. The normal urethral mucosa and the epithelial cells of the median raphe cyst usually lack melanocytes and/or melanin pigment. However, albeit extremely rare, the presence of melanin pigment and/or melanocytes in median raphe cyst, namely pigmented median raphe cyst, has been reported, although the pathogenesis of melanocytic colonization in this tumor has not been clarified (1–5). Herein, we report the sixth case of pigmented median raphe cyst and discuss the possible mechanisms of melanocytic colonization in this tumor. Written informed consent was obtained from the patient.

## Case report

### Case presentation

A 48-year-old male presented with a nodule on the ventral aspect of the penis, which had been recognized since childhood. Physical examination revealed that the nodule was slightly elevated with a smooth surface, skin-colored, elastic, soft and measured 10×3.5 mm^2^, on the median raphe of the penis. Surgical resection of the nodule was performed under the clinical diagnosis of median raphe cyst.

### Immunohistochemistry

The formalin-fixed, paraffin-embedded tissue blocks of the resected specimen were sectioned (3 μm thick), deparaffinized and rehydrated. Each section was stained with hematoxylin and eosin and then used for immunostaining. Immunohistochemical analyses were performed using an autostainer (Benchmark XT system; Ventana Medical Systems, Inc., Tucson, AZ, USA) according to the manufacturer’s instructions. The following primary antibodies were used: Mouse monoclonal antibodies against α-smooth muscle actin (alphasm-1; Novocastra Laboratories, Ltd., Newcastle upon Tyne, UK), endothelin-1 (TR.ET.48.5; Sigma-Aldrich, St. Louis, Missouri, USA), gross cystic disease fluid protein-15 (GCDFP-15; 23A3; Novocastra Laboratories, Ltd.), HMB-45 (HMB-45; Novocastra Laboratories, Ltd.), Melan-A (A103; Novocastra Laboratories, Ltd.) and stem cell factor (G-3; Santa Cruz Biotechnology, Inc., Santa Cruz, CA, USA); and a rabbit polyclonal antibody against S-100 protein (Nichirei Biosciences Inc., Tokyo, Japan).

### Histopathological and immunohistochemical results

**Figure 1:**
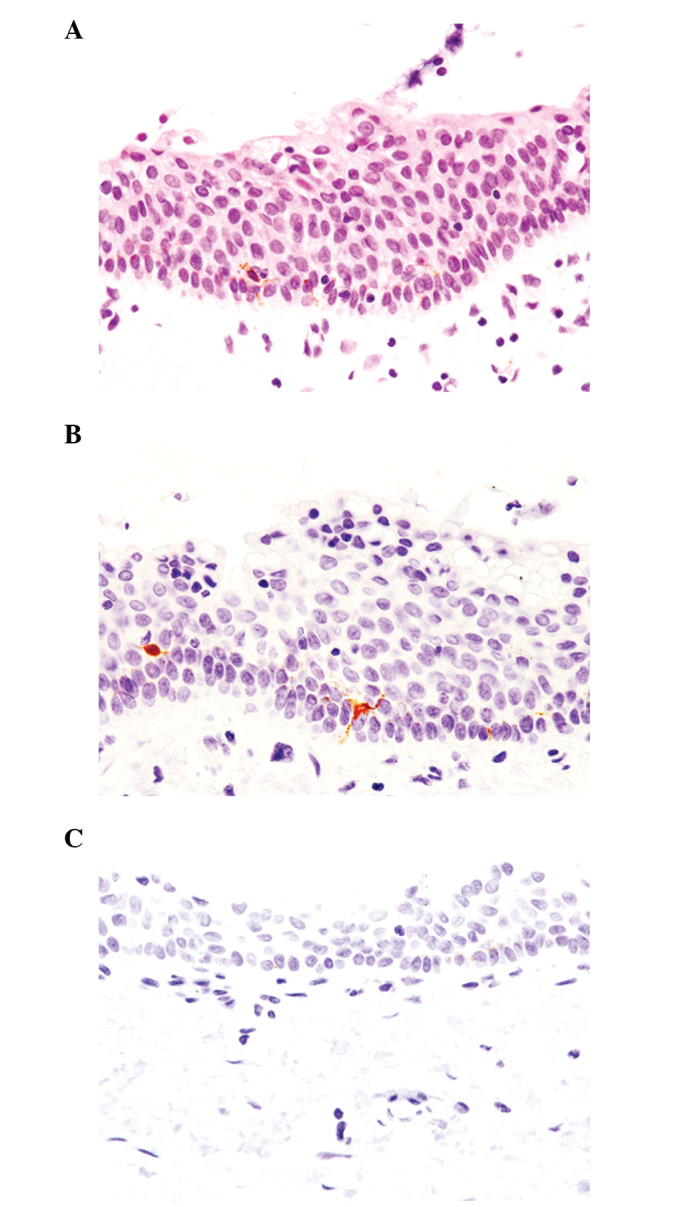
Histopathological and immunohistochemical features of the nodule on the ventral aspect of the penis. (A) The cyst wall was covered by bland urothelial cells and dendritic melanocytes were present within the epithelium. (B) Immunostaining for Melan-A clearly demonstrated dendritic melanocytes. (C) Stem cell factor was not expressed (hematoxylin and eosin; magnification, ×400).

Histopathological analysis revealed that a unilocular cyst was present under the squamous epithelium. The cyst wall was covered by two to several layers of uniform cuboidal to urothelial cells without nuclear atypia. Decapitation secretion and mucinous and ciliated cells were not observed. In addition, no mitotic figures were present. The unusual observation was the presence of dendritic melanocytes among the epithelial cells (Fig. 1A).

Immunohistochemical analyses showed that these dendritic melanocytes were positive for Melan-A and S-100 protein (Fig. 1B), but negative for HMB-45. GCDFP-15 was negative in the epithelial cells and α-smooth muscle actin-positive myoepithelial cells were not present. Moreover, stem cell factor and endothelin-1 were not expressed in the epithelial cells (Fig. 1C).

According to these histopathological and immunohistochemical results, an ultimate diagnosis of pigmented median raphe cyst was determined.

## Discussion

The cyst wall of the median raphe cyst is lined by cuboidal to columnar cells, transitional (urothelial) cells, stratified squamous cells or a mixture of these (4). The presence of mucous and ciliated cells and decapitation secretion have also been previously reported (5). Albeit rare, the presence of melanin pigment and/or melanocytes in median raphe cyst, namely pigmented median raphe cyst, has been documented (4). Previously, Nishida *et al* summarized the clinicopathological features of pigmented median raphe cyst (4). According to the analyses, the incidence of this variant is 2.1% and no clinicopathological differences have been identified between the pigmented and conventional cysts, with the exception of the presence or absence of melanin pigment and/or melanocytes (4).

It is well-known that non-neoplastic melanocytes are rarely found in various benign and malignant non-melanocytic tumors of the various sites, a phenomenon known as ‘colonization’ (6–10). The urethral mucosa is normally devoid of melanocytes and the mechanism by which melanocytes and/or melanin pigment appear in median raphe cyst remains unclear. Previously, a few hypotheses with regard to melanocytic colonization in non-melanocytic lesions have been proposed. For example, it has been suggested that the presence of melanocytes may be presented as heterotopic cell rests resulting from aberrant melanocytic migration during the embryonic period (4). It has also been hypothesized that the epithelial cells may produce factors that simulate the proliferation and differentiation of melanocytes, such as stem cell factor and endothelin-1 (11). Satomura *et al* reported a case of pigmented squamous cell carcinoma of the hard palate. The authors clearly demonstrated stem cell factor and endothelin-1 expression in the neoplastic squamous cells, which may have led to the development of melanocytic colonization in squamous cell carcinoma (11).

The current case report is the first to analyze the immunohistochemical expression of stem cell factor and endothelin-1 in median raphe cyst. It was revealed that the epithelial cells of the median raphe cyst showed negative immunoreactivity for these markers. Therefore, colonization of melanocytes in median raphe cyst is not associated with the production of stem cell factor and endothelin-1 by the epithelial cells of the cyst. In addition, aberrant migration of melanocytes may contribute to the development of this rare lesion. In conclusion, this report is the first to analyze the immunohistochemical expression of melanocytic proliferation and differentiation factors, and demonstrate that they are not involved in the pigmentation of median raphe cyst. Therefore, aberrant migration of melanocytes may contribute to the development of this rare lesion. Further analysis is required to clarify the pathogenesis of pigmented median raphe cyst.
